# Antiviral Activity of Chrysin against Influenza Virus Replication via Inhibition of Autophagy

**DOI:** 10.3390/v13071350

**Published:** 2021-07-13

**Authors:** Seong-Ryeol Kim, Myeong-Seon Jeong, Seo-Hyeon Mun, Jaewon Cho, Min-Duk Seo, Hyoungsu Kim, Jooeun Lee, Jae-Hyoung Song, Hyun-Jeong Ko

**Affiliations:** 1 Department of Pharmacy, Kangwon National University, Chuncheon 24341, Korea; ksr12134@nate.com (S.-R.K.); moonnari0606@gmail.com (S.-H.M.); hiems_nox@naver.com (J.C.); 2 Chuncheon Center, Korea Basic Science Institute (KBSI), Chuncheon 24341, Korea; jms0727@kbsi.re.kr (M.-S.J.); je1211@kbsi.re.kr (J.L.); 3 College of Pharmacy and Research Institute of Pharmaceutical Science and Technology (RIPST), Ajou University, Suwon 16499, Korea; mdseo@ajou.ac.kr (M.-D.S.); hkimajou@ajou.ac.kr (H.K.); 4 Kangwon Institute of Inclusive Technology, Kangwon National University, Chuncheon 24341, Korea

**Keywords:** chrysin, influenza A/Puerto Rico/8/34, antiviral activity, autophagy, mTOR

## Abstract

Influenza viruses cause respiratory infections in humans and animals, which have high morbidity and mortality rates. Although several drugs that inhibit viral neuraminidase are used to treat influenza infections, the emergence of resistant viruses necessitates the urgent development of new antiviral drugs. Chrysin (5,7-dihydroxyflavone) is a natural flavonoid that exhibits antiviral activity against enterovirus 71 (EV71) by inhibiting viral 3C protease activity. In this study, we evaluated the antiviral activity of chrysin against influenza A/Puerto Rico/8/34 (A/PR/8). Chrysin significantly inhibited A/PR/8-mediated cell death and the replication of A/PR/8 at concentrations up to 2 μM. Viral hemagglutinin expression was also markedly decreased by the chrysin treatment in A/PR/8-infected cells. Through the time course experiment and time-of-addition assay, we found that chrysin inhibited viral infection at the early stages of the replication cycle. Additionally, the nucleoprotein expression of A/PR/8 in A549 cells was reduced upon treatment with chrysin. Regarding the mechanism of action, we found that chrysin inhibited autophagy activation by increasing the phosphorylation of mammalian target of rapamycin (mTOR). We also confirmed a decrease in LC3B expression and LC3-positive puncta levels in A/PR/8-infected cells. These results suggest that chrysin exhibits antiviral activity by activating mTOR and inhibiting autophagy to inhibit the replication of A/PR/8 in the early stages of infection.

## 1. Introduction

Despite the increase in influenza vaccination rates, influenza A virus infection, which is the main cause of flu, continues to occur. Oseltamivir, a neuraminidase (NA) inhibitor that cleaves the sialic acid residues of the viral receptor required for releasing progeny viruses, is mainly used to treat influenza A virus infections [1]. However, infections with mutant strains of the influenza A virus that are resistant to oseltamivir are gradually emerging [2]. Owing to the high mutation rate of this virus, it can evolve rapidly and continuously, and consequently create new strains that are resistant to commercially available antiviral drugs. This emphasizes the importance of researching alternative compounds that have antiviral effects against the influenza A virus [3].

Autophagy is an evolutionarily conserved homeostatic mechanism that promotes the formation of intracellular double-membrane structures that engulf cellular components for the decomposition of unnecessary or damaged organelles [4,5]. In addition, autophagy is a part of the host defense mechanism that captures and eliminates invading pathogens, including viruses [6]. However, some viruses, including the influenza A virus, facilitate their replication by utilizing the autophagy pathway [7,8].

Chrysin (5,7-dihydroxyflavone) is a natural flavonoid that is present in honey and propolis [9], and also in several plants, such as *Passiflora caerulea* [10], *Passiflora incarnata* [11] (17966676), *Oroxylum indicum* [12,13], and *Matricaria chamomilla* [14]. Chrysin has a wide range of pharmacological and biological activities, including anti-inflammatory, anti-cancer, antibiotic, and antifungal properties [15]. In addition, our previous study showed that chrysin has antiviral activity against enterovirus 71 (EV71) and coxsackievirus B3 (CVB3) [16,17]. However, there are no studies on the antiviral activity and mode of action of chrysin against the influenza A virus. In this study, we showed that chrysin had antiviral activity against influenza A/Puerto Rico/8 H1N1 (A/PR/8) by inhibiting autophagy. In conclusion, chrysin is a potential and promising candidate as a broad-spectrum antiviral agent.

## 2. Materials and Methods

### 2.1. Viruses and Cell Lines

A/PR/8 virus was obtained from American Type Culture Collection (ATCC, Manassas, VA, USA) and propagated in A549 cells (ATCC) incubated at 37 °C in 5% CO_2_. The cells were maintained in Dulbecco’s modified Eagle’s medium (DMEM) supplemented with 10% fetal bovine serum (FBS) and 1% antibiotic–antimycotic solution (all from Gibco BRL, Invitrogen Life Technologies, Karlsruhe, Germany). TPCK-trypsin was purchased from Pierce (Thermo Fisher Scientific, Rockford, IL, USA), sulforhodamine B (SRB) and oseltamivir carboxylate were purchased from Sigma-Aldrich (St. Louis, MO, USA), and tissue culture plates were purchased from Falcon (BD Biosciences, San Jose, CA, USA). All chemicals used were of reagent grade.

### 2.2. In Vitro Assessment of Antiviral Activity

Antiviral activity was analyzed using the SRB method to analyze cytopathic effect (CPE) reduction, as previously reported [18]. Briefly, A549 cells were seeded in a 96-well plate at a density of 3 × 10^4^ cells per well and incubated for 24 h. Following this, 1 × 10^3^ pfu/90 µL of virus suspension containing TPCK-trypsin (1 µg/mL) was added to each well, along with the indicated concentration of chrysin. The cells were incubated at 37 °C in 5% CO_2_ for 3 days until an appropriate CPE was achieved. After 3 days, the A549 cells were washed with PBS and incubated for 30 min at −20 °C after the addition of ice-cold acetone (70%). After removing the acetone, the plate was dried for 30 min in a drying oven, after which, 0.4% (*w*/*v*) SRB in 1% acetic acid solution was added to each well and incubated for 30 min at 20 °C. The SRB solution was then removed and the plates were washed with 1% acetic acid before oven-drying. After drying for 1 day, SRB was solubilized with a 10 mM unbuffered Tris-based solution, and the absorbance was measured at 540 nm using a SpectraMax i3 microplate reader (Molecular Devices, Palo Alto, CA, USA) with a reference absorbance of 620 nm. The antiviral activity of each test compound in A/PR/8 virus-infected cells was calculated as a percentage of the corresponding untreated control.

### 2.3. Western Blotting

SDS-PAGE was performed as previously described [19]. Protein levels of the lysates of A/PR/8-infected A549 cells were evaluated using the following primary antibodies: mammalian target of rapamycin (mTOR), phospho-mTOR, LC3B (Cell Signaling Technology, Danvers, MA, USA), anti-influenza A virus nucleoprotein, anti-influenza A virus hemagglutinin (HA) (Abcam, Cambridge, MA, USA), and anti-β-actin (Santa Cruz Biotechnology, Dallas, TX, USA). The levels were detected using secondary antibodies, including goat anti-rabbit IgG antibody (Bio-Rad, Hercules, CA, USA), goat anti-mouse IgG F(ab’)2, and polyclonal HRP-conjugated antibody (Enzo Life Sciences, Farmingdale, NY, USA). The enhanced chemiluminescence substrate used was the femtoLUCENT™ PLUS-HRP Kit (G-biosciences, St. Louis, MO, USA). Images were obtained using the ImageQuantTM LAS 4000 mini system (GE Healthcare Life Sciences, Little Chalfont, Buckinghamshire, UK) and analyzed using ImageJ software (NIH, Bethesda, MD, USA).

### 2.4. Hemagglutination Assay

A hemagglutination assay was performed to measure the effect of chrysin on virus adsorption into cells. First, 40 µL of 2-fold serial dilution of A/PR/8 suspension was incubated with 10 µL of 50 µM and 10 µM concentrations of chrysin for 1 h at 20 °C and added to an equal volume of 2.5% chicken erythrocyte (cRBC) suspension in PBS in a round (U) bottom 96-well plate (Thermo Fisher Scientific, Waltham, MA, USA). The mixture was incubated for 2 h at 20 °C, following which, the extent of erythrocyte aggregation was measured as described previously [20].

### 2.5. Hemolysis Inhibition Assay

The inhibitory effect of chrysin on A/PR/8 fusion with host cells was analyzed using a hemolysis inhibition assay. Briefly, 100 µL of chrysin (50 µM) was mixed with an equal volume of virus-containing media and incubated at 20 °C for 30 min, following which, 200 µL of 2% cRBCs was added and incubated at 37 °C for another 30 min. Next, 100 µL each of sodium acetate (0.5 M) with pH values 4.7, 4.9, 5.1, and 5.3 was added and the mixtures were incubated at 37 °C for 30 min. The mixtures were then centrifuged at 1200 rpm for 6 min to separate intact erythrocytes, and the hemoglobin concentration in the supernatant was spectrophotometrically analyzed [20].

### 2.6. Confocal Microscopy

A549 cells (1 × 10^5^ cells/well) were seeded into 6-well culture plates. After 24 h, the cells were infected with A/PR/8 and treated with 25 μM chrysin for 24 h in DMEM containing 1 µg/mL TPCK-trypsin. The cells were washed with PBS and fixed with 4% paraformaldehyde for 10 min at 20 °C. They were then stained with primary antibodies, including anti-LC3 Abs (Cell Signaling Technology, Danvers, MA, USA), and incubated overnight at 4 °C. After washing with PBS, the cells were stained with goat anti-rabbit IgG H&L (Alexa Fluor^®^ 647) (Abcam, Cambridge, MA, USA) secondary antibodies and incubated for 2 h at 20 °C. After washing with PBS, the cells were stained with 4′,6-diamidino-2-phenylindole (DAPI) and analyzed using a confocal microscope (LSM 880 with Airyscan, Zeiss, NY, USA).

### 2.7. Quantitative Real-Time PCR

Total RNA was extracted using an RNA Extraction Mini Kit (Qiagen, Hilden, Germany). Taqman real-time PCR and reverse transcription PCR were carried out using AgPath-ID™ One-Step RT-PCR Reagents (Applied Biosystems, Waltham, MA, USA) on a Bio-Rad CFX96 thermal cycler (Bio-Rad, Hercules, CA, USA). The A/PR/8 Matrix (*M*) gene was detected using qRT-PCR. The following primers were used: M forward primer 5′- AATCCTGTCACCTCTGACTAAGG-3′, M reverse primer 5′-CATTYTGGACAAAKCGTCTACG-3′, and probe primer 5′-TGCAGTCCTCGCTCAC-3′. The following *GAPDH* primers were used: forward primer 5′-GGTCTCCTCTGACTTCAACA-3′, reverse primer 5′-AGCCAAATTCGTTGTCATAC-3′, and probe primer 5′-CCCTCAACGACCACTTTGTCAAG-3′. The cycling conditions were as follows: heating at 45 °C for 10 min for reverse transcription, reverse transcription inactivation and initial denaturation at 95 °C for 10 min, followed by 40 cycles of amplification at 95 °C for 15 s and at 60 °C for 45 s. The results were analyzed using the real-time system AB 7900HT software (Life Technologies, Carlsbad, CA, USA), and all values were normalized to *GAPDH* levels.

### 2.8. Time-of-Addition Assay

Chrysin and oseltamivir carboxylate were added to A549 cells either before (−1 h), during (0 h), or after (1, 2, 4, 6, 8, 10, and 12 h) infection with A/PR/8. For all in vitro experiments, oseltamivir carboxylate, which is the active metabolite of oseltamivir phosphate, was used. At 14 h post-infection, virus-specific *M* gene expression was analyzed using real-time PCR.

### 2.9. Time Course Experiment

A549 cells infected with A/PR/8 were harvested at 6, 8, 10, and 12 h post-infection, after which, chrysin was added. Total RNA was extracted at the indicated post-infection time points, and the level of viral RNA was analyzed using reverse transcription–real-time PCR.

### 2.10. Transmission Electron Microscopy (TEM)

A549 cells were seeded in 6-well culture plates at a density of 2 × 10^5^ cells in 2 mL of serum-containing DMEM until they reached 40–50% confluency. After 24 h, the cells were infected with A/PR/8 suspension containing 1 µg/mL of TPCK-trypsin and treated with 25 μM chrysin for 24 h. The cells were then fixed with 0.1% glutaraldehyde and 2% paraformaldehyde in phosphate buffer (pH 7.4) for 1 h at 4 °C and post-fixed with osmium tetroxide for 40 min at 4 °C. The samples were then dehydrated in a graded series of ethanol solutions, treated with a graded propylene oxide series, and embedded in Epon (TED Pella, Redding, CA, USA). Ultrathin sections of 80 nm were prepared and placed on a copper grid. The final samples were stained with uranyl acetate and lead citrate and observed using a transmission electron microscope (JEM-2100F, Akishima, Tokyo, Japan) operated at 200 kV.

## 3. Results

### 3.1. In Vitro Antiviral Activity of Chrysin against A/PR/8

**Figure 1:**
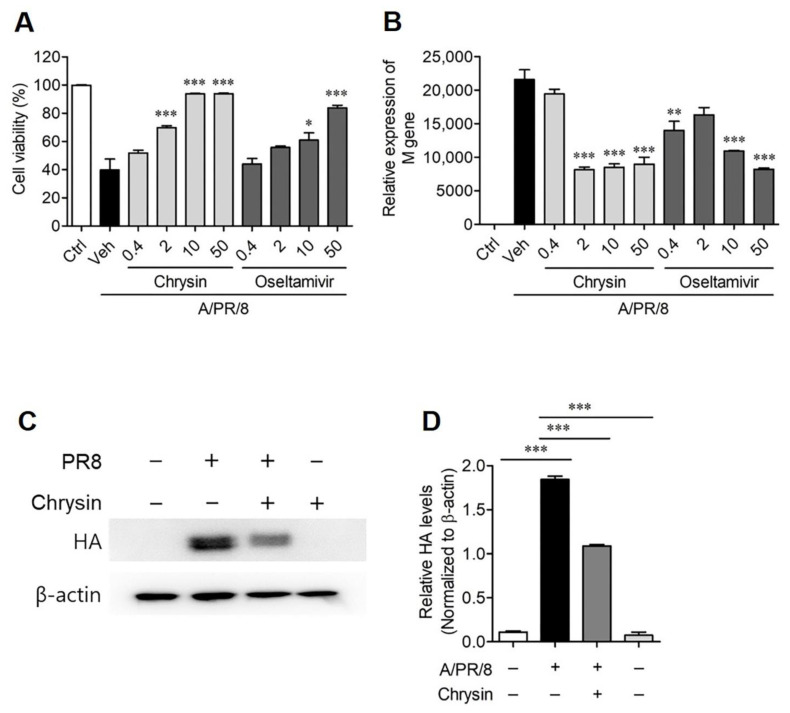
Antiviral activity of chrysin against A/PR/8 in vitro. (**A**) The antiviral activities of chrysin against A/PR/8 were investigated using A549 cells infected with 1 × 10^3^ pfu/90 µL of A/PR/8. The viability of cells was measured using an SRB assay, and the antiviral activity was calculated based on the cell viability. The results are shown as mean ± SEM. * *p* < 0.05, ** *p* < 0.01, and *** *p* < 0.001 for comparison with the A/PR/8-infected vehicle-treated group (Veh). (**B**) Relative A/PR/8 gene expression in A/PR/8-infected A549 cells was determined using real-time PCR. * *p* < 0.05, ** *p* < 0.01, and *** *p* < 0.001 for comparison with Veh based on ANOVA with Bonferroni’s multiple comparison test. (**C**) Western blot analysis of HA in A/PR/8-infected and non-infected A549 cells after treatment with the vehicle or 25 µM of chrysin for 24 h. (**D**) The ratio of HA to β-actin based on the quantification of the bands in the immunoblot, as shown in (**C**). Data are expressed as mean ± SD of the values obtained from three independent experiments carried out in triplicates. * *p* < 0.01, ** *p* < 0.001, and *** *p* < 0.0001 based on ANOVA with Bonferroni’s multiple comparison test.

Based on previous findings that showed that chrysin and its derivatives exhibit a wide range of antiviral activities against (+) ssRNA viruses belonging to the *Picornaviridae* family [16,17], we sought to determine whether chrysin has antiviral activity against other RNA viruses as well, such as the influenza virus (A/PR/8). As we assessed the virus-induced CPE in A549 cells via SRB analysis, we found that chrysin increased the survival rate of the cells that were infected with A/PR/8, whereas chrysin alone did not induce cytotoxicity (Supplementary Materials Figure S1). The A/PR/8 infection induced cell death in approximately 60% of the A549 cells, whereas treatment with 2 µM chrysin significantly prevented virus-induced cell death by exhibiting only 30% cell death (Figure 1A). We used oseltamivir carboxylate (10 µM or more) as a positive control antiviral drug that showed significant antiviral activity against A/PR/8 (Figure 1A). We also quantified the expression of the *M* gene of A/PR/8 using quantitative real-time PCR to assess the antiviral activity of chrysin. The results showed that chrysin significantly reduced the expression of the M gene, suggesting that the replication of A/PR/8 was inhibited by the chrysin treatment (Figure 1B). The inhibitory effect of chrysin on A/PR/8 replication was further confirmed via Western blotting using HA-specific antibodies. The expression level of viral HA protein in A549 cells increased 24 h after infection with A/PR/8 but showed a dramatic decrease after treatment with 25 µM chrysin (Figure 1C,D).

### 3.2. Chrysin Inhibited the Replication of A/PR/8 in the Early Stages of Viral Infection

**Figure 2:**
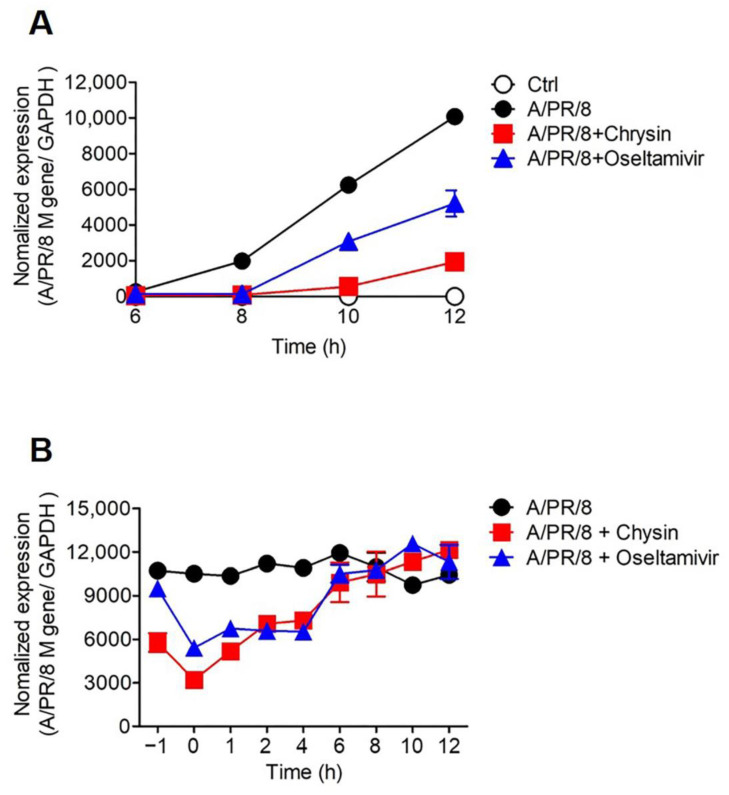
Chrysin inhibited A/PR/8 replication at an early stage of viral infection. (**A**) A549 cells infected with 1 × 10^3^ pfu/90 µL of A/PR/8 were harvested at the indicated time points, including at 6, 8, 10, and 12 h after treatment with 50 µM chrysin or 10 µM oseltamivir carboxylate. (**B**) The cells were treated with 50 µM chrysin or 10 µM oseltamivir carboxylate at the time of or after infection with the virus at the indicated time points and viral mRNA was analyzed 14 h post-infection. Total RNA was isolated and viral RNA was analyzed using reverse transcription–real-time PCR.

To analyze the antiviral mode of action of chrysin against A/PR/8, we performed time course and time-of-addition experiments. For the time course experiment, RT-qPCR analysis was conducted at 6, 8, 10, and 12 h after the A/PR/8-infected A549 cells were treated with 25 µM chrysin. The expression of the *M* gene of A/PR/8 was detected after 8 h; however, chrysin delayed the detection of viral RNA expression until 10 h after infection. Interestingly, at 10 h post-treatment, chrysin exhibited a stronger inhibition of viral RNA expression than oseltamivir carboxylate, an NA inhibitor. Therefore, we suggest that the mode of action of chrysin might be distinct from that of oseltamivir carboxylate. Based on the results of these time course experiments, we hypothesized that chrysin affected the process prior to the release of the A/PR/8 virus particles. To investigate which step was affected by chrysin, we performed a time-of-addition experiment in which 25 µM chrysin was applied to the culture medium at −1, 0, 1, 2, 4, 6, 8, 10, or 12 h post-infection, and the expression of *M* gene of A/PR/8 was analyzed at 14 h post-infection. Treatment of the A549 cells with chrysin 1 h before infection with A/PR/8 did not inhibit viral infection. However, treatment with chrysin suppressed viral RNA expression in the cells for up to 4 h after being infected by the virus. These results suggest that chrysin inhibited the early stages of the viral replication cycle (Figure 2B).

### 3.3. Chrysin Did Not Affect A/PR/8 Entry and Fusion

**Figure 3:**
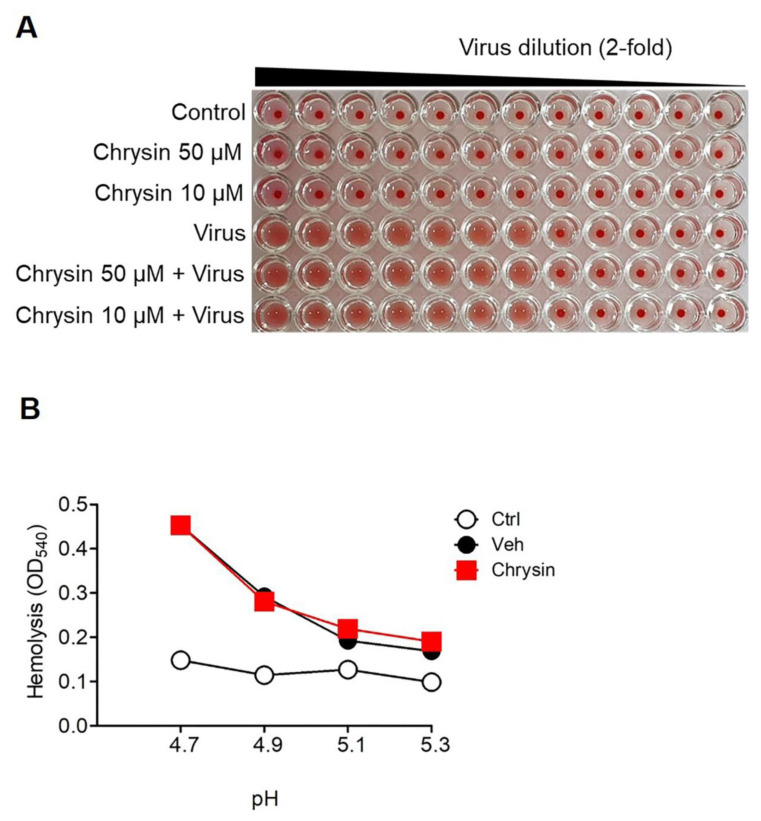
Chrysin did not inhibit the entry and fusion of A/PR/8. (**A**) Hemagglutination assay was performed as indicated in the Materials and Methods section. Chicken erythrocytes (cRBCs) were mixed with A/PR/8 in PBS and chrysin (50 µM and 10 µM) or with the vehicle (PBS containing 0.05% DMSO). (**B**) For the hemolysis inhibition assay, a mixture of chrysin (50 µM) and A/PR/8 was added to freshly prepared cRBCs. The pH condition of the mixture was adjusted to 4.7, 4.9, 5.1, and 5.3, and then incubated at 37 °C for 30 min. After a brief spin, the absorbance of the released hemoglobin contained in the supernatants was measured at 540 nm.

Next, we performed a hemagglutination assay to assess whether chrysin could interfere with the interaction between the HA present on the surface of A/PR/8 and its cellular receptor sialic acid. The inhibition of this interaction is known to prevent virus attachment to cells [21]. cRBCs were used for the hemagglutination assay; the virus concentration used in the assay was A/PR/8 titrated in a two-fold dilution in rows, starting with the highest concentration of 5 × 10^3^ pfu/40 µL. The cells were treated with 10 µL of chrysin at concentrations of 50 µM and 10 µM, and PBS was used as a negative control. A/PR/8 induced hemagglutination until diluted to 1:128, and the group treated with chrysin also exhibited hemagglutination up to the same dilution point as that of A/PR/8 (Figure 3A), suggesting that chrysin did not inhibit hemagglutination.

Next, we investigated whether chrysin interfered with the viral–cell membrane fusion mediated by the HA-2 subunit [22] by performing a hemolysis assay. The conformational changes in HA initiated the lysis of cRBCs via acidification (pHs of 4.7, 4.9, 5.1, and 5.3) of the virus–cRBC suspension, resulting in hemolysis. Wells containing no viruses were used as the control to determine the effect of the compounds on cRBCs. We found that chrysin did not inhibit A/PR/8-induced RBC hemolysis at any pH (Figure 3B). Collectively, these results suggest that the antiviral activity of chrysin was not due to the inhibition of viral attachment or viral–cell membrane fusion during the early stages of the viral life cycle of A/PR/8.

### 3.4. Chrysin Alleviated Autophagy Induced by A/PR/8 Infection

**Figure 4:**
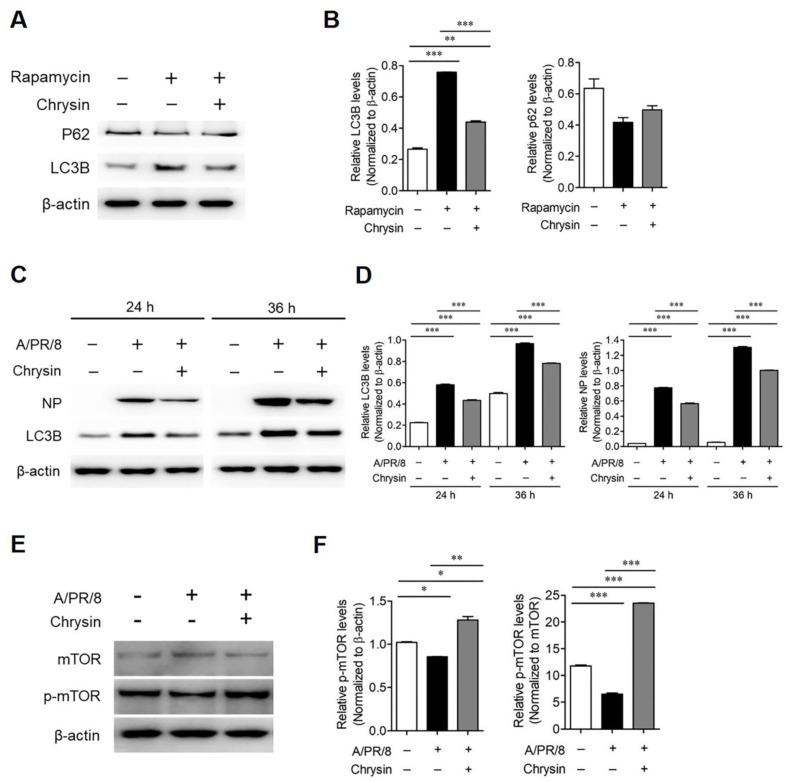
Autophagy was inhibited by chrysin in the A/PR/8-infected A549 cells. (**A**) The culture medium of A549 cells was pretreated with a control (DMSO) or 500 nM rapamycin for 1 h and was then treated with 50 µM chrysin. After 1 h, cell lysates were collected and subjected to Western blot analysis. (**B**) The ratios of LC3B to β-actin levels and p62 to β-actin levels based on the quantification of the bands in the immunoblot shown in (**A**). (**C**) Western blot analysis results of LC3B and NP in A/PR/8-infected and non-infected A549 cells after treatment with chrysin (50 µM) for 24 and 36 h. (**D**) Expression levels of LC3B and NP normalized to that of β-actin are shown in the right panel. (**E**) Western blot analysis to detect mTOR and p-mTOR in A/PR/8-infected and non-infected A549 cells after 12 h of treatment with a vehicle or 50 µM chrysin. (**F**) The ratios of p-mTOR to β-actin levels based on the quantification of the bands in the immunoblot shown in (**E**). Data are expressed as mean ± SD of the values obtained from three independent experiments. * *p* < 0.01, ** *p* < 0.001, and *** *p* < 0.0001 based on ANOVA with Bonferroni’s multiple comparison test.

**Figure 5:**
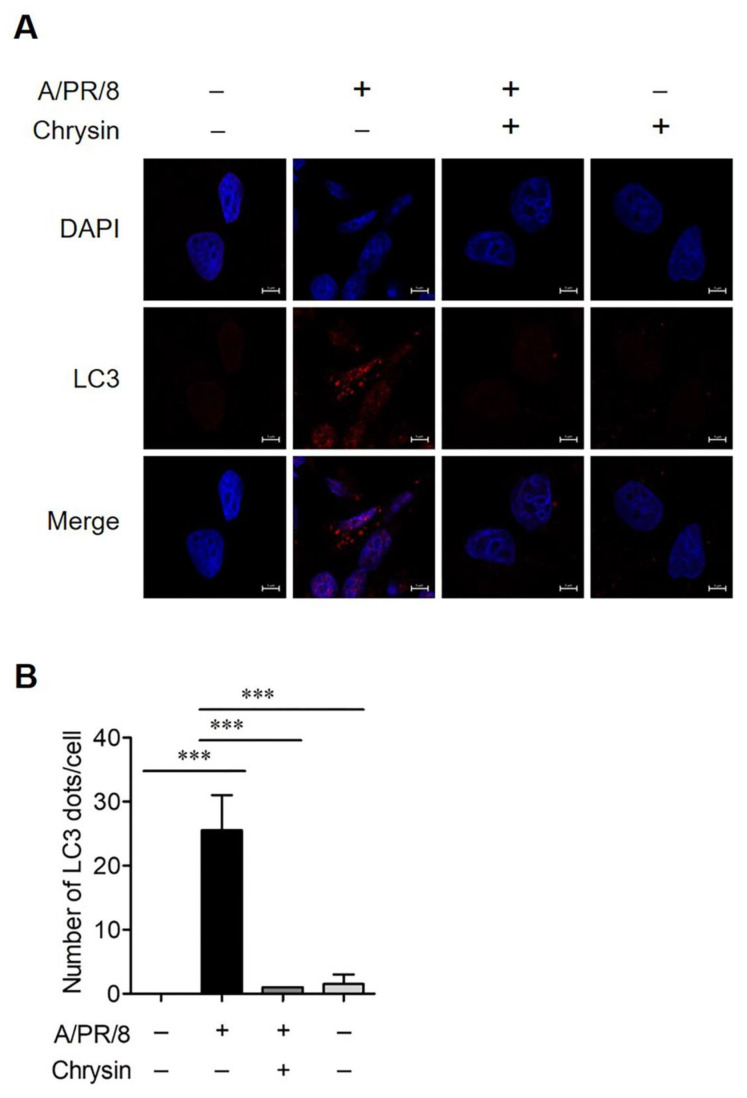
Chrysin blocked autophagy in the A/PR/8-infected A549 cells. (**A**) Representative image of LC3 staining in A/PR/8-infected and non-infected A549 cells treated with 50 µM chrysin for 24 h. The LC3 puncta was observed under a confocal microscope. Red signals represent LC3; blue signals represent DAPI-stained nuclei. Scale bar, 5 µm. (**B**) The number of RFP-LC3 dots per cell was counted. Data are expressed as mean ± SD of the values obtained from three independent experiments. *** *p* < 0.0001 based on ANOVA with Bonferroni’s multiple comparison test.

**Figure 6:**
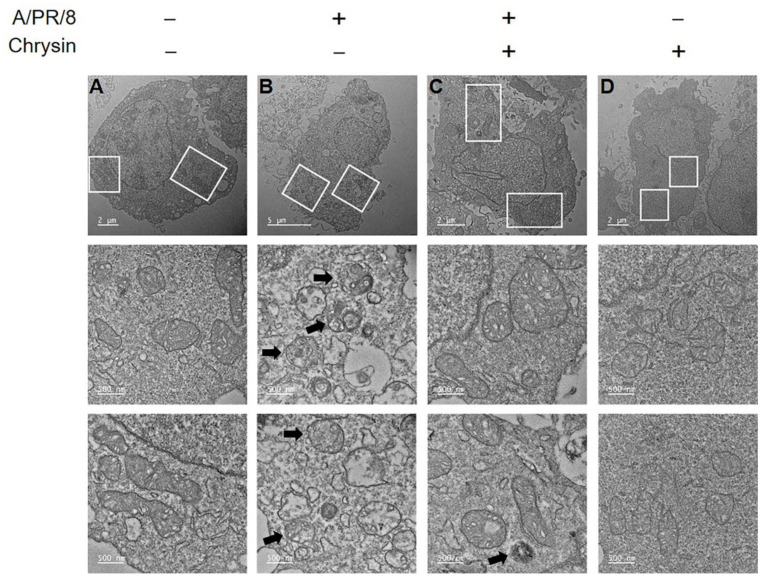
Analysis of autophagy using transmission electron microscopy (TEM). A549 cells infected with A/PR/8 were treated with 50 µM chrysin. After 24 h of treatment, the cells were rinsed with PBS, fixed, and processed for TEM. The white boxes in the upper images indicate the enlarged images shown below. The arrows indicate the sites of autophagy. (**A**) Non-infected cells. (**B**) A/PR/8-infected cells. (**C**) A/PR/8-infected cells treated with chrysin. (**D**) Non-infected cells treated with chrysin. Transmission electron micrographs at 5600× magnification are shown.

Autophagy affects the replication of many viruses, including the influenza A virus [8,23]. Recent studies have reported that viral proteins, including NS1, HA, and M2, are involved in the regulation of autophagy in influenza-infected cells [24]. Thus, we assessed whether chrysin affected autophagy activation. As a result, it was confirmed that chrysin decreased the autophagy induced by rapamycin (Figure 4A,B). Next, we evaluated whether chrysin inhibited the autophagy induced by viral infection. A/PR/8 infection in the A549 cells increased the expression of LC3B and viral NP protein at 24 h and 36 h after infection. However, interestingly, the treatment of A/PR/8-infected cells with chrysin decreased the expression of LC3B and NP proteins (Figure 4C,D). These results suggest that chrysin may suppress the virus by inhibiting autophagy. Next, we investigated whether chrysin mediates autophagy through the mTOR pathway. The level of phosphorylated mTOR was significantly higher in chrysin-treated A/PR/8-infected cells than in the untreated A/PR/8-infected cells. This suggests that chrysin inhibited autophagy by activating the mTOR pathway (Figure 4E,F). We confirmed this by performing immunofluorescence analysis on A/PR/8 virus-infected cells to assess RFP-LC3 puncta formation, which was increased in A/PR/8-infected cells and decreased in chrysin-treated infected cells (Figure 5A,B). We used TEM to confirm that double-membrane vacuoles, including autophagosomes, were induced by A/PR/8 infection and reduced by treatment with chrysin (Figure 6). Collectively, these results suggest that chrysin exhibited antiviral activity against the influenza virus in vitro by inhibiting the autophagy pathways.

## 4. Discussion

Until recently, inhibitors of NA, including oseltamivir, zanamivir, peramivir, and laninamivir, have been used to treat patients with influenza infection [25,26]. Among them, oseltamivir is the most widely used anti-influenza drug [20]. However, there has been an increase in the number of oseltamivir-resistant influenza A (H1N1) viral strains, which harbor mutations, such as the H274Y amino acid substitution [26]. Thus, the development of new antiviral drug candidates with various modes of action is necessary to overcome the resistance of the influenza virus to oseltamivir.

Autophagy, especially macroautophagy, recognizes invading microbes and forms double-membrane vesicles called autophagosomes in host cells to degrade the microbes in a lysosome-dependent manner [27]. Thus, autophagy is now recognized as a mechanism of innate immunity against pathogens that invade eukaryotic cells. In particular, autophagy also regulates interferon (IFN) signaling pathways, causing them to participate in antiviral immunity by interfering with virus infection and replication. At the same time, type I INF induces the expression of several proteins that mediate viral clearance via autophagy activation in response to virus invasion, which makes up a part of the innate immune response [28,29]. However, several viruses have evolved strategies to divert the IFN-mediated pathways and use autophagy for their own benefit [30]. Hepatitis C virus (HCV), which induces chronic latency, activates autophagy by inducing an unfolded protein response (UPR), which promotes HCV RNA replication in human hepatoma cells [31]. It is known that poliovirus and rhinovirus, both belonging to the *picornaviridae*, induce the formation of autophagy-like structures from ER membranes through intracellular infection, thereby inducing the accumulation of autophagosomes for viral RNA replication [32]. CVB3, which causes viral myocarditis, promotes viral replication by inhibiting autophagosome–lysosome fusion [33]. The viral particles produced by the Japanese encephalitis virus (JEV) are transferred to the autophagosomes for subsequent stages of viral infection [34]. In addition, a recent study reported that A/PR/8 infection simultaneously induces autophagy and Bax/caspase-dependent apoptosis and that autophagy plays a crucial role in supporting A/PR/8 replication by partially regulating virus-induced apoptosis [35].

Flavonoids have been reported to have high potential as novel antiviral agents that target several steps of the virus replication cycle [36]. In our previous study, we reported that morin hydrate, a natural flavonoid, has in vitro antiviral activity against A/PR/8 and oseltamivir-resistant A/PR/8 when used singly or in combination with oseltamivir [20]. Molecular docking studies confirmed that morin hydrate exhibits antiviral activity by binding to the HA of A/PR/8 and inhibiting the entry of A/PR/8 into host cells. In addition, in mice infected with A/PR/8, morin hydrate reduced weight loss due to viral infection and inhibited viral replication. Combination therapy with morin hydrate and oseltamivir phosphate was more effective in reducing mortality and lung inflammation in infected mice than therapy with oseltamivir phosphate alone [20]. In addition, we showed that chrysin and its derivatives showed potent antiviral activity against CVB3 in vitro, and also inhibited CVB3-associated pancreatic damage and inflammation by decreasing serum CXCL1 levels in mice [17]. Furthermore, Wang et al. demonstrated that chrysin exhibits potent antiviral activity against EV71 by inhibiting the 3Cpro protease [16].

In the current study, we evaluated the antiviral activity of chrysin against A/PR/8 and concluded that chrysin inhibited the early stages of influenza replication based on the results of time course and time-of-addition experiments. According to the study of Zhou et al., the NP and M2 protein within the influenza virus increases the expression of HSP90AA1 in a host cell, which regulates the signal pathway of AKT-mTOR in the early stage of infection, causing autophagy. Furthermore, the induced autophagy accelerates the viral polymerase activity, resulting in an increase in viral RNA synthesis and nuclear export of vRNP [8]. In addition, based on the result of the time course assay, we think that the autophagy induction by influenza virus infection may happen before 4 h post-infection. Thus, because the inhibition of autophagy in the early stages of virus infection reduces the viral burden in infected cells, we can presume that autophagy could be a novel target strategy for the development of antiviral drugs for influenza infection. Many natural flavonoids, including rutin, have been reported to activate mTOR [37], a major negative regulator of autophagy [38]. Kaempferol is known to inhibit autophagy through the decomposition of p62/SQSTM1 [39]. Likewise, we found that treatment with chrysin increased the phosphorylation of mTOR in A/PR/8-infected cells, which was otherwise decreased by A/PR/8 infection, suggesting that chrysin inhibited autophagy through the regulation of mTOR. Overall, our results suggest that chrysin exhibited antiviral activity against A/PR/8 by inhibiting autophagy induced by influenza infection, thereby suggesting that chrysin could be a novel drug candidate with a mode of action that is different from that of oseltamivir against A/PR/8 infection.

## Data Availability

The data presented in this study are available on request from the corresponding author.

## Supplementary Materials

The following are available online at https://www.mdpi.com/article/10.3390/v13071350/s1, Figure S1: Cytotoxicity of chrysin and oseltamivir in A549 cells.

## References

[1] Moscona A. (2005). Neuraminidase inhibitors for influenza. N. Engl. J. Med..

[2] Ison M.G. (2011). Antivirals and resistance: Influenza virus. Curr. Opin. Virol..

[3] Lee J.Y., Abundo M.E.C., Lee C.W. (2018). Herbal Medicines with Antiviral Activity against the Influenza Virus, a Systematic Review. Am. J. Chin. Med..

[4] Levine B., Kroemer G. (2008). Autophagy in the pathogenesis of disease. Cell.

[5] Choi Y., Bowman J.W., Jung J.U. (2018). Autophagy during viral infection—A double-edged sword. Nat. Rev. Microbiol..

[6] Senft D., Ronai Z.A. (2015). UPR, autophagy, and mitochondria crosstalk underlies the ER stress response. Trends Biochem. Sci..

[7] Dreux M., Chisari F.V. (2010). Viruses and the autophagy machinery. Cell Cycle.

[8] Wang R., Zhu Y., Zhao J., Ren C., Li P., Chen H., Jin M., Zhou H. (2019). Autophagy Promotes Replication of Influenza A Virus In Vitro. J. Virol..

[9] Schnitzler P., Neuner A., Nolkemper S., Zundel C., Nowack H., Sensch K.H., Reichling J. (2010). Antiviral activity and mode of action of propolis extracts and selected compounds. Phytother. Res. PTR.

[10] Wolfman C., Viola H., Paladini A., Dajas F., Medina J.H. (1994). Possible anxiolytic effects of chrysin, a central benzodiazepine receptor ligand isolated from *Passiflora coerulea*. Pharmacol. Biochem. Behav..

[11] Brown E., Hurd N.S., McCall S., Ceremuga T.E. (2007). Evaluation of the anxiolytic effects of chrysin, a *Passiflora incarnata* extract, in the laboratory rat. AANA J..

[12] Chen L.J., Games D.E., Jones J. (2003). Isolation and identification of four flavonoid constituents from the seeds of *Oroxylum indicum* by high-speed counter-current chromatography. J. Chromatogr. A.

[13] Harminder, Singh V., Chaudhary A.K. (2011). A Review on the Taxonomy, Ethnobotany, Chemistry and Pharmacology of *Oroxylum indicum* Vent. Indian J. Pharm. Sci..

[14] Zanoli P., Avallone R., Baraldi M. (2000). Behavioral characterisation of the flavonoids apigenin and chrysin. Fitoterapia.

[15] Kumar S., Pandey A.K. (2013). Chemistry and biological activities of flavonoids: An overview. Sci. World J..

[16] Wang J., Zhang T., Du J., Cui S., Yang F., Jin Q. (2014). Anti-enterovirus 71 effects of chrysin and its phosphate ester. PLoS ONE.

[17] Song J.H., Kwon B.E., Jang H., Kang H., Cho S., Park K., Ko H.J., Kim H. (2015). Antiviral Activity of Chrysin Derivatives against Coxsackievirus B3 in vitro and in vivo. Biomol. Ther..

[18] Hong E.H., Song J.H., Kang K.B., Sung S.H., Ko H.J., Yang H. (2015). Anti-Influenza Activity of Betulinic Acid from *Zizyphus jujuba* on Influenza A/PR/8 Virus. Biomol. Ther..

[19] Kim S.R., Song J.H., Ahn J.H., Lee G.S., Ahn H., Yoon S.I., Kang S.G., Kim P.H., Jeon S.M., Choi E.J. (2018). Antiviral and anti-inflammatory activity of budesonide against human rhinovirus infection mediated via autophagy activation. Antivir. Res..

[20] Hong E.H., Song J.H., Kim S.R., Cho J., Jeong B., Yang H., Jeong J.H., Ahn J.H., Jeong H., Kim S.E. (2020). Morin Hydrate Inhibits Influenza Virus entry into Host Cells and Has Anti-inflammatory Effect in Influenza-infected Mice. Immune Netw..

[21] Du R., Cui Q., Rong L. (2019). Competitive Cooperation of Hemagglutinin and Neuraminidase during Influenza a Virus Entry. Viruses.

[22] Kim J.I., Lee S., Lee G.Y., Park S., Bae J.Y., Heo J., Kim H.Y., Woo S.H., Lee H.U., Ahn C.A. (2019). Novel Small Molecule Targeting the Hemagglutinin Stalk of Influenza Viruses. J. Virol..

[23] Zhou Z., Jiang X., Liu D., Fan Z., Hu X., Yan J., Wang M., Gao G.F. (2009). Autophagy is involved in influenza A virus replication. Autophagy.

[24] Zhirnov O.P., Klenk H.D. (2013). Influenza A virus proteins NS1 and hemagglutinin along with M2 are involved in stimulation of autophagy in infected cells. J. Virol..

[25] Moorthy N.S., Poongavanam V., Pratheepa V. (2014). Viral M2 ion channel protein: A promising target for anti-influenza drug discovery. Mini Rev. Med. Chem..

[26] McKimm-Breschkin J.L. (2013). Influenza neuraminidase inhibitors: Antiviral action and mechanisms of resistance. Influenza Other Respir. Viruses.

[27] Espert L., Codogno P., Biard-Piechaczyk M. (2007). Involvement of autophagy in viral infections: Antiviral function and subversion by viruses. J. Mol. Med..

[28] Stark G.R., Kerr I.M., Williams B.R., Silverman R.H., Schreiber R.D. (1998). How cells respond to interferons. Ann. Rev. Biochem..

[29] Tian Y., Wang M.L., Zhao J. (2019). Crosstalk between Autophagy and Type I Interferon Responses in Innate Antiviral Immunity. Viruses.

[30] Colombo M.I. (2005). Pathogens and autophagy: Subverting to survive. Cell Death Differ..

[31] Ke P.Y., Chen S.S. (2011). Activation of the unfolded protein response and autophagy after hepatitis C virus infection suppresses innate antiviral immunity in vitro. J. Clin. Investig..

[32] Jackson W.T., Giddings T.H., Taylor M.P., Mulinyawe S., Rabinovitch M., Kopito R.R., Kirkegaard K. (2005). Subversion of cellular autophagosomal machinery by RNA viruses. PLoS Biol..

[33] Wong J., Zhang J., Si X., Gao G., Mao I., McManus B.M., Luo H. (2008). Autophagosome supports coxsackievirus B3 replication in host cells. J. Virol..

[34] Li J.K., Liang J.J., Liao C.L., Lin Y.L. (2012). Autophagy is involved in the early step of Japanese encephalitis virus infection. Microbes Infect..

[35] Yeganeh B., Ghavami S., Rahim M.N., Klonisch T., Halayko A.J., Coombs K.M. (2018). Autophagy activation is required for influenza A virus-induced apoptosis and replication. Biochim. Biophys. Acta Mol. Cell Res..

[36] Lalani S., Poh C.L. (2020). Flavonoids as Antiviral Agents for Enterovirus A71 (EV-A71). Viruses.

[37] Wang R., Sun Y., Huang H., Wang L., Chen J., Shen W. (2015). Rutin, a Natural Flavonoid Protects PC12 Cells against Sodium Nitroprusside-Induced Neurotoxicity through Activating PI3K/Akt/mTOR and ERK1/2 Pathway. Neurochem. Res..

[38] Heras-Sandoval D., Pérez-Rojas J.M., Hernández-Damián J., Pedraza-Chaverri J. (2014). The role of PI3K/AKT/mTOR pathway in the modulation of autophagy and the clearance of protein aggregates in neurodegeneration. Cell. Signal..

[39] Kim C.J., Shin S.H., Kim B.J., Kim C.H., Kim J.H., Kang H.M., Park B.S., Kim I.R. (2018). The Effects of Kaempferol-Inhibited Autophagy on Osteoclast Formation. Int. J. Mol. Sci..

