# Supplementary material for: Antiviral Activity of Chrysin against Influenza Virus Replication via Inhibition of Autophagy
Source: Viruses. 2021 Jul 13;13(7):1350. doi: 10.3390/v13071350 (PMC8310364; doi:10.3390/v13071350)
Supplement: Supplementary file 1 [file viruses-13-01350-s001.zip › viruses-1272369-supplementary.pdf]

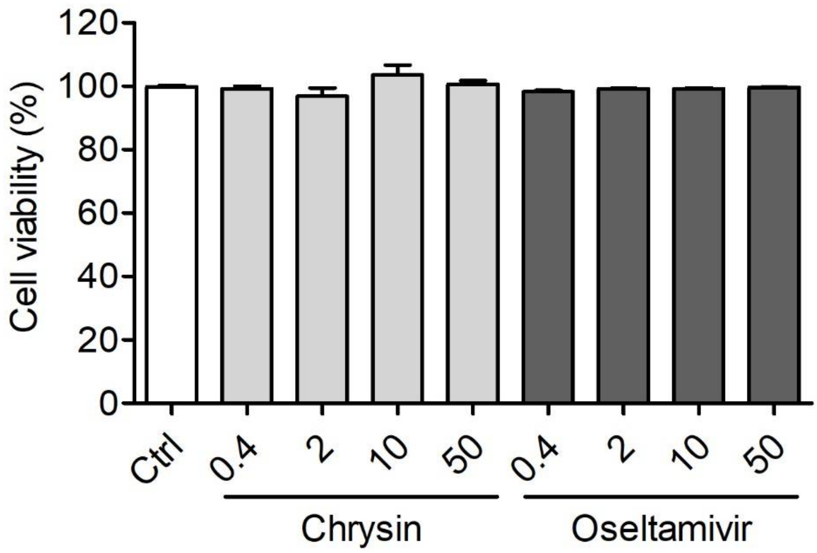

Supplementary Figure S1. Cytotoxicity of chrysin and oseltamivir in A549 cells. 3×10^4 A549 cells/well were seeded in a 96-well culture plate. On the next day, ctrl (control) was cultured with only culture media, and the chrysin and oseltamivir-treated groups were treated with chrysin and oseltamivir at concentrations of 0.4, 2, 10, and 50 μM and cultured for 2 days. Results are presented as the mean percentage values from three independent experiments, carried out in triplicates.
